# Thymoma-Associated Pure Red Cell Aplasia Following Femoral Neck Fracture

**DOI:** 10.7759/cureus.21836

**Published:** 2022-02-02

**Authors:** Morika Suzuki, Takashi Watari

**Affiliations:** 1 General Internal Medicine, National Hospital Organization Sendai Medical Center, Miyagi, JPN; 2 Hospital Medicine, University of Michigan Health System, Ann Arbor, USA; 3 Medicine, Shimane University Hospital, Izumo, JPN

**Keywords:** femoral neck fracture, anemia, thymoma-associated pure red cell aplasia, thymoma, pure red cell aplasia

## Abstract

Pure red cell aplasia (PRCA) is a rare hematopoietic disease presenting with severe anemia and a marked decrease in reticulocytes and bone marrow erythroblasts. Thymomas are the most common underlying cause of chronic PRCA and have been implicated in the development of other autoimmune diseases. However, the pathogenesis and mechanisms underlying the development of thymoma-associated PRCA remain unclear. Herein, we present a case of thymoma-associated PRCA in a patient who developed progressive anemia after a femoral neck fracture. The absence of severe anemia and the rapid progression of anemia over a two-month period suggested that the fracture and subsequent surgery may have triggered thymoma-associated PRCA. The patient was treated with cyclosporine and Primobolan but remained dependent on red blood cell transfusion.

## Introduction

Pure red cell aplasia (PRCA) is a rare hematopoietic disease characterized by normocytic normochromic anemia, reticulocytopenia, and a marked reduction in the number of bone marrow erythroblasts. Thymoma is one of the commonest underlying conditions of chronic PRCA. Thymomas may produce thymic epithelial cell (TEC)-derived autoreactive T cells, which may lead to the development of various autoimmune diseases, such as PRCA and myasthenia gravis [1]. Thymoma-associated PRCA may either be diagnosed after aetiological evaluation for PRCA or upon a close examination of the thymoma. Interestingly, PRCA may also occur after thymoma resection, although the mechanism remains poorly understood [2]. Here, we present a case of thymoma-associated PRCA diagnosed on examination of an older adult with progressive anemia after a femoral neck fracture, along with a review of relevant literature.

## Case presentation

An 88-year-old woman who was receiving medical treatment for hypertension and hyperlipidemia presented to the hospital with symptoms of anemia. Three months prior to her visit, she had suffered a femoral neck fracture due to a fall and had undergone femoral head replacement surgery at her local orthopedic clinic. Preoperative blood tests showed moderate anemia (red blood cell [RBC] count, 2.78 million/µL [3.84-5.00 million/μL]; hemoglobin, 9.4 g/dL [11.3-15.2 g/dL]) and minimal intraoperative blood loss (150 mL). Postoperative blood tests showed a temporary drop in the RBC count and hemoglobin to 2.04 million/µL and 6.8 g/dL, respectively, which recovered to 3.01 million/µL and 11.0 g/dL, respectively, without transfusion or other treatment in two weeks. However, after discharge, she began to experience shortness of breath and dizziness while performing routine activities. A blood test during a regular visit to her family physician revealed rapidly progressive anemia (RBC count, 1.44 million/µL; hemoglobin, 4.7 g/dL).

Upon arrival at our hospital, the patient had a temperature of 37.0 °C, respiratory rate of 20 breaths/ minute, blood pressure of 130/52 mmHg, pulse rate of 92 beats/ minute, oxygen saturation of 98% (room air), and pale eyelids and conjunctiva. No rales were auscultated in the respiratory tract. A systolic murmur (Levin II/VI) was heard at the tip of the heart; however, no jugular venous distension or leg edema and no signs of heart failure were observed. Mild subcutaneous bleeding at the site of femoral neck fracture surgery was noted; however, no local swelling, heat, or pain was recorded.

Blood tests showed severe anemia (erythrocytes, 1.4 million/μL; hemoglobin, 4.5 g/dL; mean corpuscular volume, 99 fL) and a marked decrease in reticulocytes (0.1%; 1,400/μL). The white blood cell (6,200 × 10³/μL) and platelet (25.2 × 10⁴/μL) counts were within normal ranges. No abnormalities in other blood counts or biochemical tests were noted.

**Figure 1:**
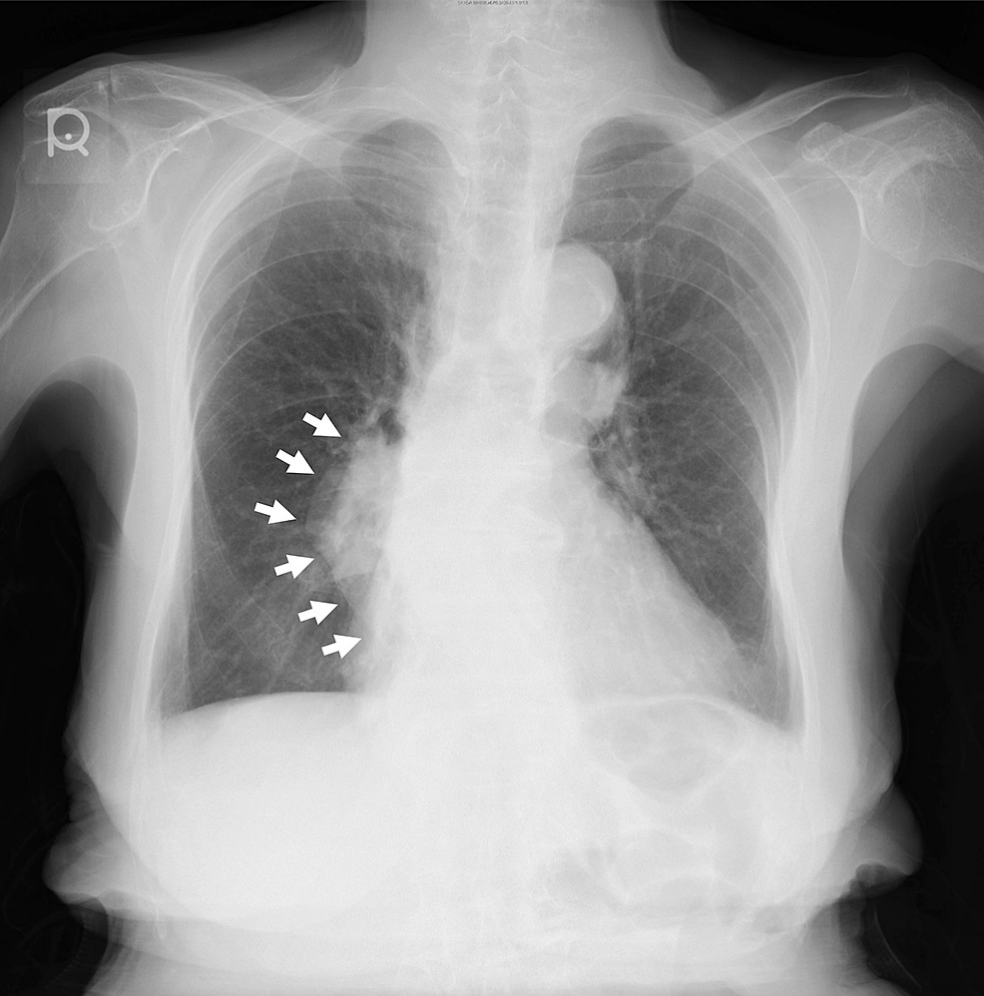
Chest radiography demonstrating abnormal shadowing and mediastinal enlargement.

The bone marrow biopsy revealed a positive myelogram with a marked decrease in erythroblastic cells to 0.2%. In addition, there was no obvious dysplasia or malignant cells in the two blood cell lines. Based on these results, PRCA was diagnosed. To determine the cause of PRCA, we confirmed negative tests for human parvovirus B19 and antinuclear antibodies, and that the patient had no history of the use of drugs, such as erythropoietin [3], which have been reported to cause PRCA. Further, no findings suggestive of leukemia, malignant lymphoma, or solid tumor invasion on bone marrow were noted. Chest radiography showed no cardiac enlargement or pleural effusion; however, a mass shadow in the right mediastinum was noted (Figure 1).

**Figure 2:**
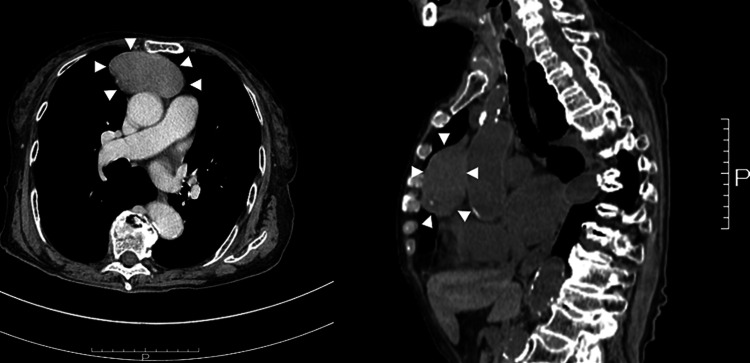
Computed tomography (CT) chest scan (left and right) showing a well-defined, 7-cm soft tissue mass in the anterior mediastinum.

Chest computed tomography showed a well-defined mass in the anterior mediastinum (Figure 2). No other findings were suggestive of solid cancer or bleeding. The fecal occult blood test was negative. Based on the test results, PRCA with thymoma was diagnosed.

Surgical resection is useful if the thymoma is malignant, but it remains unclear whether removal of the thymoma improves the PRCA. Therefore, we decided against surgical intervention because there was no obvious metastasis or invasion, and due to the patient’s age, which increases the risk of a negative outcome post-surgery. It is known that PRCA associated with thymoma responds well to cyclosporine [4]; however since the patient was an elderly post-fracture patient with osteoporosis and decreased renal function (BUN:18, Cr 0.93, eGFR 42.9, G3b), she was at risk of nephrotoxicity and osteoporosis. Thus, we treated her with reduced doses of immunosuppressive drugs (cyclosporine 2mg/kg/day) and anabolic steroids (Metenolone enanthate 5mg/day, later increased to 10mg). After three months of treatment, anemia continued to progress. The dose of cyclosporine was to be increased while monitoring the trough level, but due to the decline in renal function, the dose was not increased, and the treatment was continued with blood transfusion in parallel. Over the seven-month follow-up period, the patient remained dependent upon packed red blood cells transfusions (two units every fortnight).

## Discussion

PRCA is characterized by severe normocytic anemia, coupled with marked reticulocytopenia and erythroblastopenia, and is of two types, congenital and acquired. Acquired PRCA can be divided clinically into acute and chronic types, and pathologically into idiopathic, with no known cause, or secondary, with an underlying disease [3]. Although approximately 10% of all PRCA cases are associated with thymoma and 2%-5% of established thymoma patients present with PRCA, its pathogenetic and pathogenic mechanisms are poorly understood [3].

In contrast, several studies have suggested that CD8+ T cells may be activated via human leukocyte antigen class I and act as cytotoxic T cells to induce autoimmune disease in the bone marrow [1]. Previously, surgical resection of PRCA-associated thymomas was recommended. However, in recent years, the appearance of PRCA after removal of the thymoma has been reported [2]. Hence, it is unclear whether thymoma resection improves PRCA.

In this case, the diagnosis of PRCA complicated by thymoma was made on close examination of rapid, progressive anemia after surgery for a femoral neck fracture, suggesting that the fracture and surgery may have prompted a thymoma-associated PRCA. Generally, hemorrhage is the most suspected cause of progressive postoperative anemia; however, in this case, the reticulocyte count was not elevated and there was no hematopoiesis, leading to the PRCA diagnosis. The mechanism by which thymomas trigger PRCA is unknown; however, reportedly, stress-induced glucocorticoids stimulate TECs to induce thymus atrophy and improve stress release [5]. Possibly, TECs may also be affected, thereby leading to increased activity of cytotoxic T cells and causing PRCA.

Currently, there are no case reports on advanced anemia after a non-thymoma-related surgery that resulted in a diagnosis of thymoma-associated PRCA; however, we believe that more reports may contribute to the understanding of the pathogenesis of PRCA.

## Conclusions

PRCA is a rare hematopoietic disease in which only erythropoiesis is suppressed, whereas white blood cell and platelet maturation are normal. In thymoma cases, thymoma-associated PRCA is an autoimmune-mediated change in the bone marrow. Furthermore, thymoma-associated PRCA can be triggered by stress. Therefore, physicians should consider the combination of the above diseases as a differential diagnosis for anemia.
